# Intestinal Antisepsis by Purgation

**Published:** 1897-01

**Authors:** 


Intestinal Antisepsis by Purgation. To a healthy man whose fecal matter contained 68,000 germs per milligramme and who eliminated thus about 12,000 millions of microbes per day, Gilbert and Dominici gave a purgative composed of 15 grains of sulphate of soda and 15 grains of sulphate of magnesia. The stools emitted under its influence contained 273,253 microbes per milligramme, and the total number eliminated during the day amounted to 411,000 millions. The purgative action continuing the next day produced a diarrhœtic stool containing 55,000 germs per milligramme, in all nearly 20,000 millions. The following day the feces regained their normal character ; they contained only 1350 microbes per milligramme—in all a little more than 500 millions. The purgative had then brought about an asepsis (state of freedom from putrefaction) of the digestive canal, if not absolute, at least remarkable. The authors consider it probable that the purgation, by increasing the number of microbes in the fecal matter, increase likewise the virulence of these matters, because the microbes of the small intestine are more virulent than those of the large. If, then, the increase in the number and virulence of the intestinal microbes can occasion diarrhœa, inversely diarrhœa increases the number and virulence of the germs in the excrement. It appears to be certain that the asepsis following purgation is of only short duration. The authors had shown previously that a milk-diet is capable of bringing about an almost absolute asepsis in the digestive canal. This property of milk is owing to the fact that it is completely absorbed, leaves no residue, and furnishes no food for microbes. The action of milk is less rapid than that of purgatives, and only becomes evident at the end of five hours, but is persistent and lasts as long as the milk-diet is continued.—*Annales de Méd. Vét.*, March, 1896.

